# College Notes

**Published:** 1894-05

**Authors:** 


### College Notes.

THE forty-ninth annual commencement of the Medical Department of the University of Buffalo will be held on Tuesday, May 1, 1894. The Alumni Association will hold its business session in Alumni Hall at 10.30 A. M. The afternoon session will be devoted to the reading of scientific papers, as follows: The Treatment of Several

Common Ailments and Particularly the Relationship Between Croupous Pneumonia as an Infectious Process and its Therapeutics, by H. A. Hare, M. D., professor of therapeutics in the Jefferson Medical College; The Medical Examination of the Living Human Body when Required by Courts, by Tracy C. Becker, Esq., president New York State Bar Association and legal editor of a Treatise on Medical Jurisprudence, Forensic Medicine and Toxicology; The Etiology, Pathology and Treatment of Certain Phases of Gonorrhea, by Dr. George Emerson Brewer, assistant demonstrator of anatomy, College of Physicians and Surgeons, New York; and also a paper by Dr. Charles G. Stockton, subject not announced.

At 7.30 P. M. the commencement exercises will be held at Music Hall, after which the annual banquet will be held. Members and friends of the University and alumni association are requested to be present and participate in the various exercises.

---

DR. WILLIAM PEPPER, for many years provost of the University of Pennsylvania, resigned that office April 23, 1894. He felt that the great growth of the university during his administration demanded the undivided efforts of the provost. He, however, will continue to hold the professorship of medicine. Dr. Pepper signalized his retirement from office by a contribution of $50,000, that will be applied to the extension of the university hospital buildings.

---

THE commencement exercises of the Medical Department of Niagara University will be held at the Buffalo Academy of Music on Wednesday evening, May 9, 1894, at 8 o'clock, to which the general public is invited.

